# Annotations

**Published:** 1910-01-15

**Authors:** 


### ANNOTATIONS.

### The Late Dr. Thomas Savill.

The Algiers correspondent of the *Daily Mail* has cabled the news of the death, by accident, of Dr. Thomas Dixon Savill, who was spending a winter holiday in Morocco, and whose name is well known in connection with nervous diseases. Dr. Savill received his professional education at St. Thomas's and St. Mary's Hospitals, and passed several terms in graduate study at Hamburg, Paris, and Vienna, where his name was familiar to those interested in his speciality. He graduated M.B. with honours at the London University in 1881 and a year later took his M.D., and some years later the D.P.H. of Cambridge. He was for a time in general practice and then returned to St. Thomas's, where he filled the usual house appointments. Later on he was elected Assistant Physician and Pathologist at the West London Hospital, member of the Anatomical Society of Paris and Honorary President of the Société des Sciences Medicales d'Angers. He was physician to the West-End Hospital for Diseases of the Nervous System and to the Hospital for Diseases of the Skin, Leicester Square, and the author of many monographs and textbooks. As a post-graduate teacher he was very popular; his afternoon clinics at the West-End Hospital were always well attended and were made doubly interesting by his geniality and wide range of sympathy, which enabled him to deal with his patients in a singularly gentle fashion. No less were his demonstrations at the West London Hospital, when he was attached to it, appreciated by his graduate pupils, and equally thorough were his lectures at the St. John's Hospital. He was a cultured and widely-read man, a good linguist, and with his interests and sympathies not wholly bound up in his speciality only, but keenly alive to extra professional interests as well. He was a frequent and valued contributor to The Hospital, with which he was associated for many years, and was a colleague with whom it was a real pleasure to work. We tender our sincere condolences to his wife and family at this sudden and unexpected blow.

### Ankylostomiasis and National Character.

A year or so ago Hellenic scholars and others were deeply interested by the appearance of a monograph wherein an ingenious author sought to prove that the decay of Greece, which set in about the beginning of the fourth century before Christ, was due to the importation of malaria into the swampy valleys in which a large proportion of the population dwelt. Plausible and ingenious as were the arguments by which this thesis was supported, it by no means secured universal acceptance; but if it has served to attract public attention to the profound degree in which matters of public health and hygiene may modify the fate of a nation, the determination of its exact truth is of comparatively small moment. Fortunately an instance is available of the way in which national temperament may be affected by the prevalence of disease, which can be more readily investigated than can be problems of hæmatozoology in ancient Greece. In a series of reports issued within the third quarter of this year by the Public Health Service of the United States the prevalence of ankylostomiasis in the Southern States is described; and it is not easy to resist the conclusions drawn that the laziness and shiftlessness which have long been recognised as an essential part of the character of the "poor white" are really due to the anæmia produced by the ravages of this parasite. The reports include a vivid description of the filthy and insanitary habits of the Southern peasantry, both white and black, and tables from prison medical officers showing how enormously death-rates can be reduced when in a limited and controllable community a definite campaign for the eradication of the ankylostomum is undertaken. Another inference to which Dr. C. W. Stiles inclines strongly is that the great mortality from pulmonary tuberculosis is partly due to the diminution of resisting power caused by malarial anæmia. This mortality is more marked among the negroes, who suffer less from the parasite directly than do whites, probably owing to inherited immunity; but it is thought that the balance is redressed indirectly through the medium of the bacillus tuberculosis. A point which it might interest a military historian to consider is to what extent the issue of the American Civil War was determined by ankylostomiasis. If the population of the Southern States is infected and affected to the extent Dr. Stiles believes, the triumph of the Northerners, which was effected only with the greatest difficulty, may perhaps have been rendered possible by this same agency.

### The Gas Fire.

There is a great deal of mistaken prejudice against the gas fire. Probably owing to its experience in times past with obsolete types of gas fires, the public has come to regard this useful domestic comfort as utterly insanitary and unhygienic. The recent inquests into the deaths of persons killed through breathing impure gases generated by badly-fitted gas fires in inadequately ventilated rooms have still further tended to strengthen the prejudice against the gas fire. Nowadays, however, there are such excellent patterns in the market, the gas fire can be so beautifully fitted, and its advantages in the way of cleanliness, convenience, ease of control, and satisfactory consumption of impurities are so great that it is again winning favour. From a hygienic point of view, the modern gas fire of the type now used in hospitals, for instance, is irreproachable. Provided with a proper flue and safeguarded with a correct screen, it is admirably suited for public and private rooms, and is a perfectly safe and healthy method of heating. Its one drawback is that it consumes a greater quantity of oxygen than does the ordinary coal fire, and for that reason it is imperative that rooms in which such fires are installed should be well ventilated.

